# Supplementary material for: One-Stage Hydrothermal Growth and Characterization of Epitaxial LaMnO3 Films on SrTiO3 Substrate
Source: Materials (Basel). 2022 Aug 27;15(17):5928. doi: 10.3390/ma15175928 (PMC9457439; doi:10.3390/ma15175928)
Supplement: Supplementary file 1 [file materials-15-05928-s001.zip › materials-1876678-supplementary.pdf]

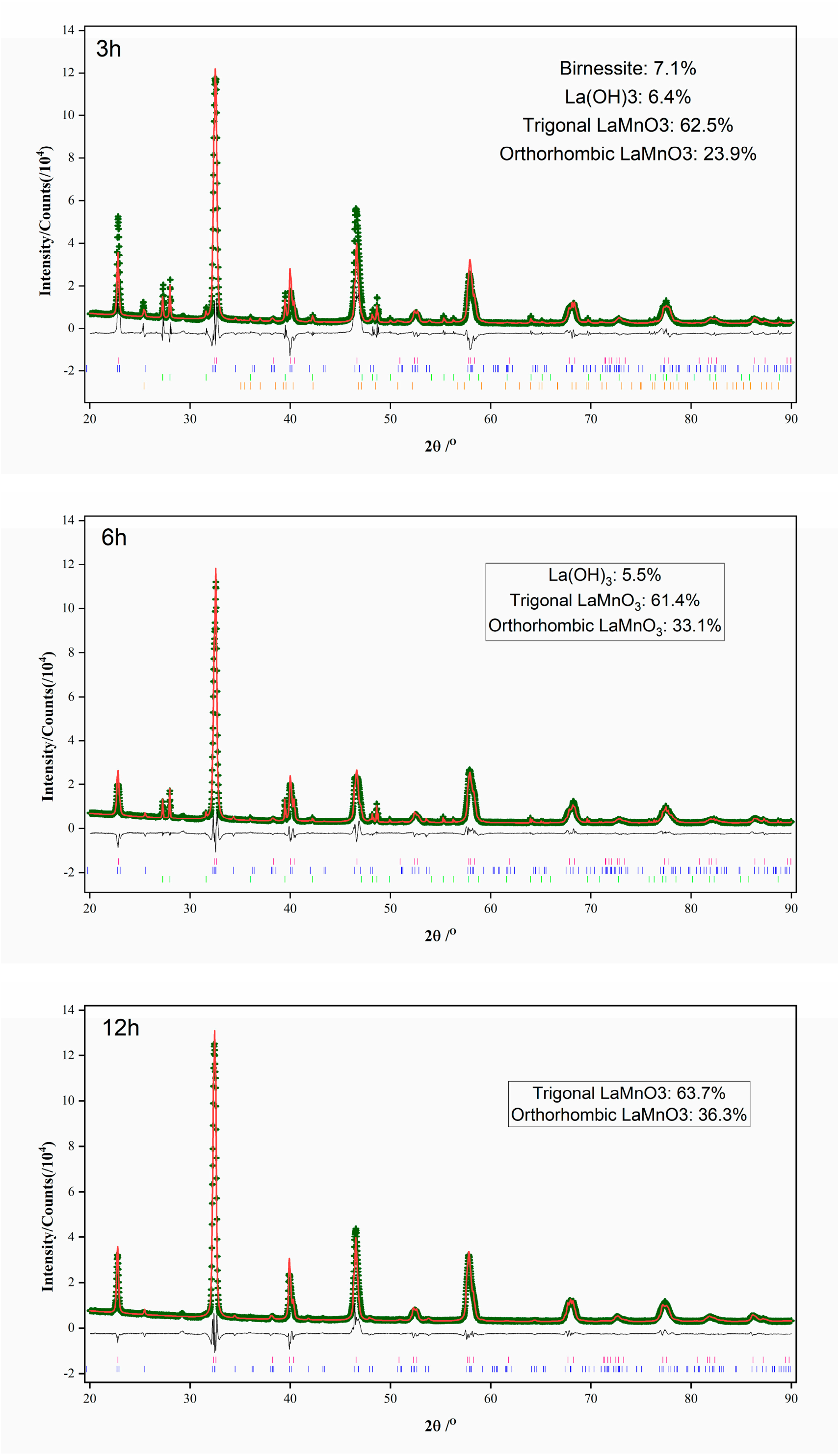

Figure S1 Rietveld phase quantitative analysis of the $LaMnO_3$ powder at 3,6 and 12h. The experimental powder X-ray diffraction data (black + marks), calculated powder X-ray diffraction data (black solid line) and the difference between experimental and calculated data (black lower line) are shown. marks the reflection positions for trigonal (red, upper) and orthorhombic (blue, lower) $LaMnO_3$.

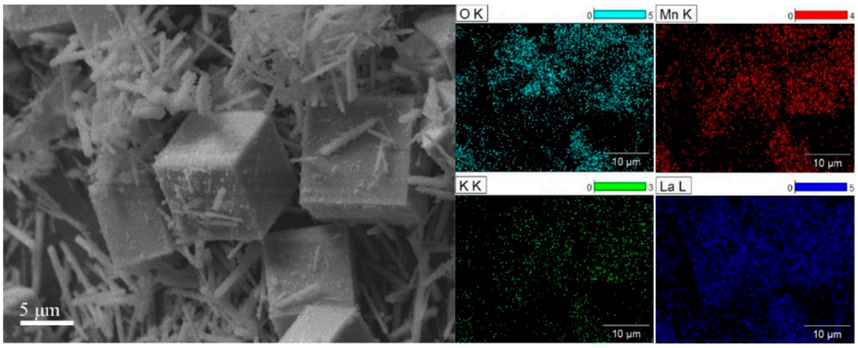

Figure S2 SEM image of hydrothermal product at 2h together with EDS elemental mapping. All elements are distributed uniformly across the sample surface.
